# Analysis of the association of single nucleotide polymorphisms of interleukin-23 receptor (IL-23R) and inflammatory bowel disease in a Chinese Han cohort

**DOI:** 10.18632/oncotarget.12296

**Published:** 2016-09-27

**Authors:** Zhong-Kai Lu, Zhi-Rong Chen, Jun-Yi Zhu, Ya Xu, Xian Hua

**Affiliations:** ^1^ Department of Gastroenterology, Suzhou Municipal Hospital (Eastern), Suzhou Hospital Affiliated to Nanjing Medical University, Suzhou, China

**Keywords:** interleukin-23 receptor, single nucleotide polymorphisms, inflammatory bowel disease, ulcerative colitis, Crohn's disease, Pathology Section

## Abstract

Inflammatory bowel disease (IBD) is a chronic, complex genetic disease with rapidly increasing prevalence in China. The interactions of genetic, environmental, and microbial factors contribute to the development of IBD, however, the precise etiologies of IBD are not well understood yet. Interleukin-23 receptor (IL-23R) encodes a subunit of receptor for IL-23, which is an important proinflammatory cytokine. In this study, we investigated the relationship between the single nucleotide polymorphism (SNP) of IL-23R gene and IBD in Chinese Han population. We genotyped three nonsynonymous IL-23R SNPs with amino acid changes (rs11209026, p.Arg381Gln; rs41313262 p.Val362Ile and rs11465797 p.Thr175Asn) in 198 patients with IBD (124 UC and 74 CD) and 100 healthy controls. The prevalence of the A allele in IL-23R Arg381Gln of CD appeared less than controls, but it was not statistically significant (2.70% *vs*. 6.00%, *p* > 0.05). There was no statistical difference between UC and controls (5.65% *vs.* 6.00%, *p* = 0.91). The p.Val362Ile variant was present in 2.42% of UC patients, in 2.70% of CD patients, which was similar in the control (2.00%). There was no statistical difference among these three groups. We did not detect Thr175Asn (rs11465797 c.524 C>A) in all the three groups. In conclusion, our study demonstrated that the p.Val362Ile and Arg381Gln were not associated with susceptibility to IBD in Chinese Han population.

## INTRODUCTION

The two major subtypes of inflammatory bowel disease (IBD), including ulcerative colitis (UC) and Crohn's disease (CD), are relapsing, chronic inflammatory disorders mainly affecting gastrointestinal track [1, 2]. Typical UC symptoms are in presence of diarrhea, bloody stool with pus, while the common symptoms of CD are abdominal pain, especial in lower right area, diarrhea and weight loss [3, 4]. Recent epidemiological studies revealed that the incidence of IBD has been progressively rising in Eastern Europe, South America, and Asia, including China, India and Japan, once low incidence areas [5-8]. The increasing incidence of IBD causes a major and growing economic and social burden to these countries.

The exact etiologies of IBD remain elusive, but evidence indicates that genetic factors play an important role in the pathogenesis of IBD [1, 2, 7]. The advent of innovative genomic technologies, especially oligonucleotide array-based genotyping (microarray) and next-generation sequencing (NGS), empower us to delineate the genetic susceptibility of complex diseases in a unprecedentedly unbiased and “hypothesis-free” manner [9]. For example, high-throughput genome-wide association studies (GWASs) had successfully identified nearly 100 genetic loci (genes) associated with IBD [1, 10, 11]. These genes centered on several pathways that are vital for intestinal homeostasis, including innate immunity (activation of NFkB pathway induced by Toll-like receptors, TLRs and a cluster of intracellular bacterial sensors, NODs), autophagy and endoplasmic reticulum (ER) stress (ATG16L1, ATG5, IRGM, NOD2, XBP1), adaptive immunity (IL-23R, JAK2, TYK2, STAT3, IL-12B), barrier dysfunction (ECM1, CDH1, HNF4A) [1, 7, 12-14].

Among the pathways discussed above, IL-23R/IL-17 axis is salient for the pathogenesis of IBD because this axis bridges the innate and adaptive immune systems [15, 16]. Interleukin-23 receptor (IL-23R) gene is mapped on chromosome 1p31, encoding the proinflammatory cytokine interleukin-23 that participates in gastrointestinal overactive inflammation [17]. IL-23 is a pivotal cytokine that works on autoimmune diseases by regulating T helper cell differentiation and is involved in the pathogenesis of susceptible IBD patients [18-20]. Previous studies have identified a significant association between IL-23R SNP and CD. Genome-wide analysis demonstrated that the Arg381Gln of IL-23R gene polymorphisms is associated with CD in non-Jewish [21]. But, subsequent studies in different countries draw different conclusions [17, 21-29]. This discrepancy implies the relationship between the Arg381Gln and the onset of CD needs further study. Over the last ten years, the studies of IBD genetics have burgeoned in both eastern and western countries and has deepen our understanding of the pathogenesis of IBD. At the same time, we also witnessed a significantly ethnic differences in IBD frequency and genetic predisposition of IBD between Asians and Caucasians. Our work aimed to explore the association between IL-23R SNPs, including Arg381Gln and another two non-synonymous variants Val362Ile and Thr175Asn, and IBD in Chinese Han population [30].

## RESULTS

### Clinical features of patients

The IBD group included 124 cases of UC and 74 cases of CD with the median age of 48 and 37, respectively. All the patients were free of other confounding auto-immune diseases. In these UC patients, 29.03% (36/124) had entire large bowel involvement and 70.97% had local colorectal involvement. Of the CD patients 16.22% had isolated small intestinal disease, 32.43% had both small intestine and large bowel involvement and 51.35% had local or entire large bowel disease only. The clinical characteristics of patients with CD or UC were given in Table 1 and Table 2, respectively.

**Table 1:** The demographic data and clinical features of the patients with CD.

Total number	74
Sex (male/female)	32/42
Median age (yr)	37
Disease localization	
Ileal	12 (16.2%)
Colon	38 (51.4)
Ileocolon	24 (32.4%)
Disease behavior	
Nonstricturing,nonpenetrating	35 (47.3%)
Stricturing	23 (31.1%
Penetrating	16 (21.6%)
Upper GI tract	4 (5.4%)
Perianal	21 (28.4%)
Extraintestinal manifestations	11 (14.9%)
Family history of IBD	17 (23.0%)
History of surgical intervention	26 (35.1%)

**Table 2:** The demographic data and clinical features of the patients with UC.

Total number	124
Sex (male/female)	63/61
Median age (yr)	48
Disease localization	
Local large bowel	88 (71.0%)
Entire large bowel	36 (29.0%)
Extra intestinal manifestations	24 (19.4%)
Family history of IBD21	(16.9%)
History of surgical intervention	14 (11.3%)

### The SNPs of IL-23R analysis

To assess the contribution of IL-23R SNPs to IBD susceptibility, we targeted three nonsynonymous SNPs: rs11209026 (c.1142G>A, p.Arg381Gln), rs41313262 (c.1084 G>A p.Val362Ile) and rs11465797 (c.524 C>A, p.Thr175Asn). The three SNPs were all demonstrated a single-peaked homozygosis genotype. The allele frequencies of the three SNPs examined in IBD patients and healthy controls are shown in Table 3. The allele G/A was expressed in rs11209026. The genotype of p.Arg381Gln variant (allele A) was present in 6.00% of healthy controls and 2.70% of CD patients [p** = 0.509, odds ratio (*OR*) = 0.435], and in 5.65% of UC (p** = 0.91, *OR* = 0.937). The overall genotype distribution of rs11209026 SNP appeared different between CD and healthy controls; however it did not reach statistical significance (p > 0.05). UC patients showed very similar frequency of rs11209026 A allele compared to healthy controls.

Despite of the low genotype frequencies of Arg381Gln in CD compared to UC, we found no significant genotype-phenotype correlations regarding to gender, age disease site and life style. The allele G/A was also expressed in rs41313262. The frequency of the p.Val362Ile variant (allele A) was very low, but comparable in IBD patients (2.42% in UC and 2.70% in CD) and controls (2.00%). The allele C was expressed in rs11465797. The p.Thr175Asn variant (allele A) was not observed in IBD patients and controls. And also there was no correlation between the genotype and phenotype respectively in IBD.

**Table 3:** The single nucleotide polymorphisms of IL-23 receptor in inflammatory bowel disease and controls.

Risk factor	Control *n*(%)	UC *n*(%)	CD *n*(%)	UC *vs* control		CD *vs* control	
				*P* value	OR	*P* value	OR
**Total**	**100**	**124**	**74**	**-**	**-**	**-**	**-**
SNPs:							
rs11209026(Arg381Gln) :							
	6 (6.00%)	7 (5.65%)	2 (2.70%)	0.91	0.937	0.051	0.435
rs41313262(Val362Ile):							
	2 (2.00%)	3 (2.42%)	2 (2.70%)	1.00	1.215	1.00	1.361
rs11465797(Thr175Asn)							
	0 (0%)	0 (0%)	0 (0%)	-	-	-	-

rs11209026 (c.1142G>A, p.Arg381Gln), rs41313262(c.1084 G>A p.Val362Ile) and rs11465797(c.524 C>A p.Thr175Asn); *OR,* odds ratio.

## DISCUSSION

IBD has been characterized as a complex disease induced by multiple interactions among environmental factors, individual genetic background [31]. Increasing evidence suggests that genetic factors play an important role in pathogenesis of IBD [1]. Previous study had demonstrated that the concordance rate of CD incidence in enzygotic twins is 40%∼60%, 5%∼20% of UC [12]. And the risk of incidence will increase 15-fold if the first degree relative has IBD [12]. The increased incidence between identical twins and the familial risk of IBD further confirms the genetic factors in the pathogenesis of IBD [14]. Searching for the predisposing genes of IBD becomes one of the most intensive research areas in gastroenterology.

IL-23 is one of the well-known and key cytokines contributing to autoimmune disorders in animal models and human. IL-23 binds the heterodimeric IL-23R complex [17], resulting in non-specific inflammatory reaction through IL-23/IL-23R/Th17/IL-17 signal transduction pathway [17]. IL-23 stimulates Th17 regulatory cells to produce IL-17 and IL-6, which induces IBD by causing the tissue damage [16, 25, 32]. The single nucleotide polymorphism is one of the most commonly inheritable variants and accounts 90% of polymorphism [33]. The nonsynonymous SNPs are of clinic importance because it changes not only amino acid sequence, but also the structure of the coding protein. In this study, the allele G of IL-23R is replaced by A in p.Arg381Gln and the amino acid Arginine (Arg) changes to glutamine (Gln). The Gln is semi-necessary amino acid for gut mucosa metabolism and participates in regulating the gastrointestinal immune. The Gln decreases intestinal permeability and preserves gut mucosa integrity, thus protecting the gut mucosal barrier function [34]. The low expression of p.Arg381Gln might play an important role in the dysfunction of gut mucosal barrier as demonstrated in non-Jewish population [21]. Other studies further confirmed the relationship of p.Arg381Gln with CD in non-Jewish, Canadian children and Italian population [22-24]. Tremelling and co-works also replicated this result in IBD patients from UK [27]. But Arg381Gln perhaps possesses intensive transmission unbalance in CD families. The effect of IL-23R SNP may have genetic heterogeneity in different ethnic groups. Yamazaki and colleagues studied the relationship of Arg381Gln with CD in Japanese, and their result showed that there was no association between Arg381Gln and CD [29]. The study of Venegas revealed that Arg381Gln was not associated with CD either in Chile population either [28]. To our knowledge, this study is the first one to investigate the relationship of Arg381Gln with IBD in Chinese Han population. Our results demonstrate that the Arg381Gln in the IL-23R gene did not protect from CD or UC in Chinese Han population. Furthermore, although it was not supported by statistical power, it seems that the low expression of Arg381Gln prefers to CD compared with UC (2.7% VS 5.6%). This observation should be further addressed in a larger samples of IBD patients.

Another two nonsynonymous SNPs rs41313262 (c.1084 G>A p.Val362Ile) and rs11465797(c.524 C>A p.Thr175Asn) also change the amino acid code . Whether the SNPs of Val362Ile and Thr175Asn will cause any disease is still unknown. Our results revealed that the polymorphism existed in site rs41313262 of IL-23R, including the p.Val362Ile variant in 2.42% of UC patients and in 2.70% of CD patients. The frequency of allele in IBD patients was similar in the control (2.00%), which indicated there was no association with IBD in Chinese Han population. In addition, we demonstrated that rs11465797 (c.524 C>A p.Thr175Asn) was not present in Chinese Han population, probably due to the genetic heterogeneity in different ethnic groups. Other possible explanation of absence of SNP in rs11465797 of IL-23R is that the gut mucosa metabolism and immune have no obvious association with valine(Val)/isoleucine(Ile)and threonine(Thr)/asparagine(Asn).

In conclusion, among the three IL-23R SNPs studied, Arg381Gln and Val362Ile are present, but not associated with IBD in Chinese Han population. Further deep, mechanistic investigations are required to delineate predisposing genes of IBD in Chinese Han population.

## MATERIALS AND METHODS

### Patients and samples

We enrolled a total of 298 participants: 124 patients with UC and 74 patients with CD, as well as 100 healthy controls. All the patients were diagnosed based on Vienna classification and managed in the Suzhou Hospital Affiliated to Nanjing Medical University in Suzhou, China. All participants were composed of only one ethnic group (Chinese Han group). Patients affected with other concomitant autoimmune diseases such as asthma, rheumatoid arthritis, systemic lupus erythematosus were excluded. Related clinical features and data of these patients were also collected and summarized in Table 1 and 2. All healthy controls had no gastrointestinal symptoms and had normal complete blood count and normal biochemical profile.

Informed consent was obtained from all the participants or their guardians for genetic testing. The research project was conducted according to the Declaration of Helsinki and was approved by the Institutional Review Board (IRB) of our hospital.

### Sample preparation and DNA extraction

Whole peripheral blood (PB) was collected from each subject. Mononuclear cells were separated by using Ficoll-Paque (Sigma, Saint Louis, MO, USA) density gradient centrifugation. Genomic DNA was extracted from PB mononuclear cell using the Tiangen DNA extraction kit (Tiangen Biotech Co. Ltd, Beijing, China) according to the recommended procedure. The concentration and purity of DNA product were determined by its optical absorbance and were diluted to 50 ng/μL.

### Analysis of IL-23R SNPs

Polymerase chain reaction (PCR) amplification of IL-23R was carried out to obtain DNA fragments containing SNPs (rs11209026, rs41313262 and rs11465797). Primers were designed for PCR based on the reference sequence GenBank NM_144701.2 (NIH, Maryland, USA) of IL-23R. The primer (synthesized by Sangon Biotech Co. Ltd, Shanghai, China) for rs11209026 and rs41313262 was forward: 5′-GAGCAGAGTAAAGAGAATAGT AA-3′; reverse: 5′-TGGG CTGAG GACTTAGCCTCTTTAAGCCTC-3′. The primer for rs11465797 was as previously described [30]: forward: 5′-CAAGTAACTGGGATTACAGGCACATG-3′; reverse: 5′-CTTTA CCTATATCATCCAGGTG-3′. DNA templates were amplified using the following PCR conditions: 95°C for 1 minute; 33 cycles of 95°C for 30 seconds; 60°C for 30 seconds; 72°C for 30 seconds and final 72°C for 5 minutes. The amplified DNA product was 461 bps and 380 bps, respectively. In the sequencing process, the DNA products were purified and the conditions of sequencing reaction: 96°C for 10 seconds; 25 cycles of 96°C for 10 seconds; 50°C for 5 seconds; 60°C for 4 minutes; 60°C for 4 minutes; store at 4°C. Sequencing primer for rs11209026 and rs41313262 was 5′-GAGCAGAGTAAAGAGAATAGTAA-3′ and bilateral sequencing for rs11465797 because of the gliding movement of T base in its forward direction sequencing (Naxin Biotech Co.Ltd, Shanghai, China). The Sequencing results were analyzed with Chromas 1.62 software (Technelysium Pty Ltd, Helensvale, Australia).

### Statistical analysis

The allele frequencies were calculated and statistical analysis was performed by SPSS 13.0 software (SPSS Inc, Chicago, USA). Proportions and categorical variables across groups were compared using χ2 test to find the difference of SNP frequencies. *P* values below 0.05 were considered to be significant for SNPs.
